# Effect of Apolipoprotein E ε4 on the association between health behaviors and cognitive function in late midlife

**DOI:** 10.1186/1750-1326-5-23

**Published:** 2010-06-01

**Authors:** Séverine Sabia, Mika Kivimaki, Meena Kumari, Martin J Shipley, Archana Singh-Manoux

**Affiliations:** 1 INSERM, U1018 Centre for Research in Epidemiology & Population Health, Hopital Paul Brousse, 16 avenue Paul Vaillant Couturier, Bâtiment 15/16, F-94807, Villejuif, France; 2 Department of Epidemiology and Public Health, University College London, UK; 3 Centre de Gérontologie, Hôpital Ste Perine, AP-HP, Paris, France

## Abstract

The extent to which the effect of risk factors on cognitive ageing is dependent on APOE ε4 remains unclear. The objective of this study is to examine whether APOE ε4 allele modifies the association between health behaviors and cognition in late midlife. Data are drawn from 5447 participants of the Whitehall II study, health behaviors were assessed in 1997-1999 (mean age = 55.6, Standard Deviation (SD) = 6.0) and APOE genotype and cognitive function in 2002-2004 (mean age = 60.9, SD = 5.9). Among APOE ε4 non-carriers, current smokers had lower scores on memory (difference in T-score = -2.49, 95%CI: -3.37, -1.60), reasoning (-2.88, 95%CI: -3.74, -2.01), phonemic (-2.66, 95%CI: -3.56, -1.76) and semantic (-2.38, 95%CI: -3.28, -1.47) fluency compared to never smokers. In APOE ε4 carriers, difference between current and never smokers was seen only for reasoning (-1.92, 95%CI: -3.31, -0.51). Interaction terms supported differential effects of smoking as a function of APOE ε4 status for memory (*p *= 0.01), and phonemic (*p *= 0.008) and semantic fluency (*p *= 0.02). Cognitive scores were lower among non-drinkers compared to moderate drinkers, among the sedentary participants and those who ate fruits and vegetable less than 2 times per day irrespective of APOE ε4 status. This study suggests that the APOE ε4 allele modifies the association of smoking but not that of other health behaviors - alcohol consumption, physical activity, fruit and vegetable consumption - with cognitive function in late midlife.

## Findings

Carriers of the apolipoprotein E (APOE) ε4 allele are at increased risk of late onset Alzheimer's disease [1]. Among APOE ε4 carriers there is evidence of atypical brain activity in young adulthood [2] and greater cognitive decline in middle-age [3,4]. Recently, it has been suggested that, in addition to these direct associations, APOE ε4 might also influence cognition indirectly by modifying the effect of non-genetic risk factors, such as health behaviors, on cognitive ageing [5,6]. Indeed, adverse health behaviors are risk factors for impaired cognition and dementia [5,7]; however, a recent review concluded that more research is needed to confirm or refute the hypothesis of a differential effect of health behaviors among APOE ε4 carriers [7]. Thus, the objective of the present study is to examine whether APOE ε4 allele modifies the association of smoking, alcohol consumption, physical activity and dietary behavior with cognitive function in late midlife.

Data are drawn from the Whitehall II study, established in 1985 on 10 308 British civil servants (6895 men and 3413 women) [8]. The University College London ethics committee approved the study and all participants gave written consent to participate in the study. The study population for the present analysis is composed of 5447 participants with complete data on health behaviors assessed during 1997-1999 (wave 5 of the study, mean age = 55.6, Standard Deviation (SD) = 6.0) and APOE genotype and cognitive function assessed during 2002-2004 (wave 7 of the study, mean age = 60.9 (SD = 5.9)).

**APOE genotype **Two TaqMan assays (Rs429358 and Rs7412, Assay-On-Demand, Applied Biosystems) were used and run on a 7900HT analyzer (Applied Biosystems), and genotypes indicated by the Sequence Detection Software version 2.0 (Applied Biosystems). Each SNP was found to be within Hardy Weinberg Equilibrium (χ2 p > 0.4). Genotyping was repeated for 511 participants and error rates were found to be < 0.15%. APOE ε2/ε3/ε4 haplotypes were created and participants were categorized as APOE ε4 non-carriers and APOE ε4 carriers for those with at least one ε4 allele.

**Health behaviors **were assessed via questionnaire *Smoking status *was assessed using questions on current and past smoking and categorized as never, past and current smokers. *Alcohol consumption *was assessed via questions on the number of alcoholic drinks ("measures" of spirits, "glasses" of wine, and "pints" of beer) consumed in the last seven days, converted to number of units of alcohol with each unit corresponding to 8 grams of ethanol. Alcohol consumption was categorized as: abstinence (none), moderate (1-14 units/week for women and 1-21 units/week for men), and high consumption (15 or more units/week in women and 22 or more in men). *Physical activity *questionnaire included 20 items on frequency and duration of participation in different physical activities (e.g. walking, sports, and gardening) that were used to compute hours per week of moderate and vigorous physical activity. This information was used to identify those with *sedentary habit*, defined as less than one hour per week of moderate physical activity and less than one hour per week of vigorous physical activity. *Dietary behavior *was assessed using a measure of *frequency of fruit and vegetable consumption *with the question "How often do you eat fresh fruit or vegetables?"; responses were on an eight-point scale, ranging from 'seldom or never' to 'two or more times a day'.

**Cognitive function **was measured with multiple tests *Short-term verbal memory *was assessed with a 20-word free recall test. Participants were presented a list of 20 one or two syllable words at two second intervals and then had two minutes to recall in writing as many of the words in any order. The *AH4-I *(Alice Heim 4-I) was used to assess *reasoning*. This test is composed of a series of 65 verbal and mathematical reasoning items of increasing difficulty [9]. It tests inductive reasoning, measuring the ability to identify patterns and infer principles and rules. Participants had 10 minutes to do this section. We used two measures of *verbal fluency*: *phonemic and semantic *[10]. Phonemic fluency was assessed via "S" words and semantic fluency via "animal" words. Subjects were asked to write as many words beginning with "S" and as many animal names as they could. One minute was allowed for each fluency test.

**Covariates **in the analysis, assessed alongside the health behaviors, were sex, age, socioeconomic position indexed as 6-level British civil service employment grade [8], and education (no or lower primary school, lower secondary school, higher secondary school, university, and higher university degree).

All cognitive scores were standardized to T-scores which are similar to z-scores but have a mean of 50 and a SD of 10 in order to allow comparison between tests. The association between categories of health behaviors and cognitive T-scores was examined using analysis of covariance (ANCOVA). The difference in mean cognitive T-score, as well as the 95% confidence intervals (CI), adjusted for age and sex, was calculated across health behavior categories separately for APOE ε4 non-carriers and carriers. In order to examine whether the APOE ε4 allele modifies the association between health behaviors and each cognitive measure, an interaction term between APOE ε4 status and health behavior categories was included in the model containing the main effects, separately for each health behavior. We also used multiple analysis of covariance (MANCOVA) to assess the overall interaction between each health behavior and APOE ε4 status for all cognitive measures considered together. The use of MANCOVA serves two purposes; it takes the correlation between the cognitive tests into account and provides a *p *value for the overall interaction term [11]. This method reduces the likelihood of Type I error, common when multiple outcomes are analysed separately. Finally, in order to examine whether the results were robust to the effects of socioeconomic position, all analyses were repeated with additional adjustment for employment grade and education.

Table 1 presents the characteristics of the participants as a function of their APOE ε4 status. Among the 3929 non-carriers, 14.9% were smokers, 12.5% were alcohol abstainers, 26.4% were sedentary, and 39.6% ate fruit and vegetables less than 2 times per day. The corresponding figures among the 1518 APOE ε4 carriers were no different, 14.6%, 14.1%, 25.7%, and 41.5%, respectively.

**Table 1:** Characteristics of the study population.

	APOE ε4 non-carriers	APOE ε4 carriers
N	3929	1518

Age (M, SD)	60.9 (5.9)	60.8 (5.9)

Women (N, %)	1071 (27.3)	384 (25.3)

Lower employment grade (N, %)	839 (21.4)	312 (20.6)

Less than secondary school (N, %)	1641 (41.8)	634 (41.8)

**Smoking status (N, %)**		
Current	587 (14.9%)	221 (14.6%)
Past	1468 (37.4%)	560 (36.9%)
Never	1874 (47.7%)	737 (48.6%)

**Alcohol consumption (N, %)**		
Abstinence	490 (12.5%)	214 (14.1%)
Moderate	2016 (51.3%)	749 (49.3%)
High	1423 (36.2%)	555 (36.6%)

**Sedentary habit (N, %)**		
Yes	1039 (26.4%)	390 (25.7%)
No	2890 (73.6%)	1128 (74.3%)

**Fruit & vegetable consumption less than 2 times/day (N, %)**		
Yes	1555 (39,6%)	630 (41,5%)
No	2374 (60,4%)	888 (58,5%)

The associations between health behaviors in 1997-1999 and cognitive function in 2002-2004 as a function of the APOE ε4 allele are presented in Table 2. Among the APOE ε4 non-carriers, current smokers had lower scores on memory (difference in T-score = -2.49, 95%CI: -3.37, -1.60), reasoning (-2.88, 95%CI: -3.74, -2.01), phonemic (-2.66, 95%CI: -3.56, -1.76) and semantic (-2.38, 95%CI: -3.28, -1.47) fluency compared to never smokers. In the APOE ε4 carriers, a corresponding difference between current and never smokers was only seen for reasoning (-1.92, 95%CI: -3.31, -0.51). Interaction terms for memory (*p *= 0.01), phonemic (*p *= 0.008) and semantic fluency (*p *= 0.02) supported differential effects of smoking as a function of APOE ε4 status. Compared to never smokers, ex-smokers had on average better scores in phonemic (+1.35, 95%CI: 0.27, 2.42) and semantic (+1.27, 95%CI: 0.25, 2.30) fluency among the APOE ε4 carriers but not among the non-carriers (*p *for interaction = 0.04 and 0.006, respectively). MANCOVA also suggested an overall interaction between APOE ε4 status and both current (*p *= 0.04) and past smoking (*p *= 0.02).

**Table 2:** Association between health behaviors and cognitive function as a function of APOE ε4 status adjusted for socioeconomic variables.

	Memory	Reasoning	Phonemic fluency	Semantic fluency	***p *for interaction**^‡^
	
Health behaviors	Difference^**† **^(95% CI)	Difference^**† **^(95% CI)	Difference^**† **^(95% CI)	Difference^**† **^(95% CI)	
**Current smoking**^§^					
APOE ε4 non-carriers	-2.49 (-3.37, -1.60)*	-2.88 (-3.74, -2.01)*	-2.66 (-3.56, -1.76)*	-2.38 (-3.28, -1.47)*	
APOE ε4 carriers	-0.34 (-1.79, 1.11)	-1.92 (-3.31, -0.51)*	-0.35 (-1.82, 1.11)	-0.34 (-1.74, 1.06)	
*p for interaction*	*0.01*	*0.24*	*0.008*	*0.02*	*0.04*
**Past smoking**^§^					
APOE ε4 non-carriers	-0.21 (-0.89, 0.44)	-0.44 (-1.08, 0.20)	0.05 (-0.62, 0.72)	-0.39 (-1.06, 0.28)	
APOE ε4 carriers	0.71 (-0.35, 1.78)	0.08 (-0.95, 1.11)	1.35 (0.27, 2.42)*	1.27 (0.25, 2.30)*	
*p for interaction*	*0.11*	*0.36*	*0.04*	*0.006*	*0.02*

**Alcohol abstinence**^#^					
APOE ε4 non-carriers	-1.24 (-2.18, -0.30)*	-3.80 (-4.71, -2.90)*	-3.05 (-4.00, -2.10)*	-2.57 (-3.52, -1.61)*	
APOE ε4 carriers	-2.16 (-3.62, -0.71)*	-3.04 (-4.44, -1.63)*	-2.88 (-4.34, -1.41)*	-3.06 (-4.46, -1.66)*	
*p for interaction*	*0.22*	*0.43*	*0.88*	*0.48*	*0.37*
**High alcohol consumption**^#^					
APOE ε4 non-carriers	0.63 (-0.08, 1.34)	1.15 (0.47, 1.84)*	1.13 (0.41, 1.85)*	1.19 (0.47, 1.91)*	
APOE ε4 carriers	1.19 (0.05, 2.32)*	0.90 (-0.19, 1.99)	1.96 (0.82, 3.10)*	0.77 (-0.33, 1.86)	
*p for interaction*	*0.39*	*0.73*	*0.23*	*0.55*	*0.31*

Sedentary habit**					
APOE ε4 non-carriers	-0.90 (-1.55, -0.20)*	-1.20 (-1.90, -0.50)*	-1.50 (-2.23, -0.80)*	-1.50 (-2.18, -0.80)*	
APOE ε4 carriers	-0.60 (-1.73, 0.53)	-1.30 (-2.43, -0.30)*	-1.30 (-2.46, -0.20)*	-2.00 (-3.12, -1.00)*	
*p for interaction*	*0.92*	*0.72*	*0.90*	*0.25*	*0.70*

**Fruit & vegetable consumption less than 2 times/day**^**††**^					
APOE ε4 non-carriers	-1.50 (-2.15, -0.90)*	-3.20 (-3.83, -2.60)*	-2.10 (-2.75, -1.50)*	-3.10 (-3.73, -2.50)*	
APOE ε4 carriers	-1.20 (-2.16, -0.20)*	-3.00 (-3.92, -2.00)*	-1.30 (-2.29, -0.30)*	-2.70 (-3.67, -1.80)*	
*p for interaction*	*0.45*	*0.59*	*0.15*	*0.46*	*0.69*

* p < 0.05.

^**† **^Mean difference in T-score of cognitive function in models adjusted for age and sex. ^‡ ^*p *from MANCOVA.

^§^Difference with never smoking. ^# ^Difference with moderate alcohol consumption.

** Difference with non sedentary habit. ^**†† **^Difference with the group eating fruit & vegetables 2 or more times a day.

Cognitive scores were lower among non-drinkers compared to moderate drinkers, among the sedentary participants and those who ate fruit and vegetables less than 2 times per day. These associations were present in both APOE ε4 non-carriers and carriers with no evidence of differential effects as a function of the APOE ε4 status (*p *for interaction from MANCOVA was 0.37 for alcohol abstinence, 0.31 for high alcohol consumption, 0.70 for sedentary habit, and 0.69 for fruit and vegetable consumption). In models further adjusted for employment grade and education (Table 3), the effect sizes were somewhat attenuated in both APOE ε4 carriers and non-carriers. However, the interaction terms remain unchanged except that for past smoking.

**Table 3:** Association between health behaviors and cognitive function as a function of APOE ε4 status adjusted for socioeconomic variables.

	Memory	Reasoning	Phonemic fluency	Semantic fluency	***p *for interaction**^‡^
	
Health behaviors	Difference^**† **^(95% CI)	Difference^**† **^(95% CI)	Difference^**† **^(95% CI)	Difference^**† **^(95% CI)	
**Current smoking**^§^					
APOE ε4 non-carriers	-1.65 (-2.53, -0.79)*	-1.10 (-1.84, -0.36)*	-1.46 (-2.32 -0.61)*	-0.99 (-1.83, -0.14)*	
APOE ε4 carriers	0.41 (-1.02, 1.84)	-0.36 (-1.59, 0.86)	0.73 (-0.67, 2.12)	0.88 (-0.42, 2.17)	
*p for interaction*	*0.01*	*0.27*	*0.009*	*0.02*	*0.02*
**Past smoking**^§^					
APOE ε4 non-carriers	0.17 (-0.47, 0.82)	0.28 (-0.26, 0.83)	0.56 (-0.07, 1.19)	0.19 (-0.42, 0.82)	
APOE ε4 carriers	0.76 (-0.28, 1.80)	0.15 (-0.75, 1.04)	1.40 (0.38, 2.42)*	1.30 (0.35, 2.25)*	
*p for interaction*	*0.30*	*0.68*	*0.22*	*0.06*	*0.17*

**Alcohol abstinence**^#^					
APOE ε4 non-carriers	-0.12 (-1.06, 0.81)	-1.07 (-1.86, -0.28)*	-1.21 (-2.13, -0.30)*	-0.51 (-1.40, 0.39)	
APOE ε4 carriers	-1.56 (-3.00, -0.12)*	-1.65 (-2.88, -0.42)*	-2.00 (-3.40, -0.59)*	-1.97 (-3.28, -0.66)*	
*p for interaction*	*0.08*	*0.67*	*0.58*	*0.09*	*0.28*
**High alcohol consumption**^#^					
APOE ε4 non-carriers	0.25 (-0.45, 0.94)	0.33 (-0.26, 0.91)	0.55 (-0.13, 1.23)	0.53 (-0.14, 1.20)	
APOE ε4 carriers	0.91 (-0.20, 2.03)	0.16 (-0.80, 1.11)	1.49 (0.40, 2.58)*	0.15 (-0.86, 1.17)	
*p for interaction*	*0.37*	*0.62*	*0.23*	*0.48*	*0.30*

Sedentary habit**					
APOE ε4 non-carriers	-0.37 (-1.05, 0.32)	0.06 (-0.52, 0.64)	-0.66 (-1.33, -0.00)*	-0.52 (-1.18, 0.14)	
APOE ε4 carriers	-0.03 (-1.15, 1.09)	-0.08 (-1.03, 0.88)	-0.52 (-1.61, 0.57)	-1.14 (-2.15, -0.12)*	
*p for interaction*	*0.81*	*0.88*	*0.64*	*0.35*	*0.71*

**Fruit & vegetable consumption less than 2 times/day**^**††**^					
APOE ε4 non-carriers	-0.60 (-1.22, 0.01)	-1.12 (-1.65, -0.60)*	-0.65 (-1.26, -0.05)*	-1.49 (-2.08, -0.90)*	
APOE ε4 carriers	-0.49 (-1.47, 0.50)	-1.43 (-2.27, -0.59)*	-0.19 (-1.16, 0.77)	-1.53 (-2.43, -0.64)*	
*p for interaction*	*0.70*	*0.74*	*0.37*	*0.94*	*0.82*

* p < 0.05.

^**† **^Mean difference in T-score of cognitive function in models adjusted for age, sex, employment grade, and education. ^‡ ^*p *from MANCOVA.

^§^Difference with never smoking. ^# ^Difference with moderate alcohol consumption.

** Difference with non sedentary habit. ^**†† **^Difference with the group eating fruit & vegetables 2 or more times a day.

These prospective data from over five thousand British adults suggest that the APOE ε4 allele modifies the impact of smoking but not that of other health behaviors - alcohol consumption, physical activity, fruit and vegetable consumption - on cognitive function in late midlife. Among the APOE ε4 non-carriers, current smokers had lower scores on multiple cognitive tests, such as memory, phonemic and semantic fluency, compared with never smokers. A corresponding pattern was not observed among the APOE ε4 carriers.

Our findings are in agreement with previous studies on clinical outcomes which documented evidence of an interaction between APOE ε4 and current smoking. In those studies, a higher risk of dementia among smokers was found, but only among APOE ε4 non-carriers [12-16]. We also observed higher cognitive scores among ex-smokers compared to never smokers among the APOE ε4 carriers. A previous study reported similar results for the risk of Alzheimer's disease [12]. However, in our analysis the effect of past smoking, compared with never smoking, did not differ as a function of APOE ε4 status when socio-economic position was taken into account. Thus, the higher scores among the ex-smokers who were APOE ε4 carriers compared to APOE ε4 non-carriers appeared to be due to confounding by socioeconomic factors.

It is plausible that the effects of smoking in APOE ε4 carriers are attenuated as these individuals have been shown to have fewer nicotinic receptor binding sites and lower activity of choline acetyltransferase than non carriers [17]. Smoking might counterbalance the deficit in nicotine receptors and the lack of acetylcholine found in APOE ε4 carriers [14,18]. Another possible mechanism behind the smaller impact of smoking in APOE ε4 carriers relates to several processes associated with the APOE ε4 allele that accelerate degenerative changes in arteries and brain, such as a somewhat higher total blood cholesterol, more oxidized blood lipids, and greater risk of coronary heart disease [2,19,20]. Such genetic influences may reduce the relative importance of other risk factors for cognitive ageing, such as smoking. Given this, it is surprising that there was no clear evidence of effect modification by APOE ε4 on the association between other health behaviors and cognition. Our results confirm previous studies suggesting that the effect of health behaviors on cognition does not differ by APOE ε4 status for alcohol [21-23], physical activity [21,24,25], or diet [26].

The strengths of this study include a detailed assessment of health behaviors and a large battery of cognitive tests covering multiple cognitive domains. However, although our sample covered a wide socioeconomic range, with annual full-time salaries ranging from £4,995 to £150,000, the data are exclusively from white-collar civil servants and cannot therefore be assumed to be representative of the general population. The use of multiple testing increases the risk of Type 1 error and in order to ensure the robustness of our findings we repeated the analyses using MANCOVA to obtain an overall interaction term. The conclusions were similar from both analyses. In conclusion, this study suggests that smoking is associated with lower cognitive function only among APOE ε4 non-carriers. Presence of the APOE ε4 allele did not modify the impact of other health behaviors on cognition.

## List of abbreviations

APOE: Apolipoprotein E; ANCOVA: analysis of covariance; MANCOVA: multiple analysis of covariance; AH4-I: Alice Heim 4-I

## Conflict of interests

The authors declare that they have no competing interests.

## Authors' contributions

SS analysed the data and wrote the first full draft of the paper. All authors jointly designed the hypothesis, interpreted the data and edited the paper. SS had full access to all of the data in the study and takes responsibility for the integrity of the data and the accuracy of the data analysis. All authors read and approved the final manuscript.
